# Epidural Contrast Patterns and Clinical Implications: An Educational Review

**DOI:** 10.1007/s11916-025-01396-x

**Published:** 2025-05-14

**Authors:** Giuliano Lo Bianco, Barnabas T. Shiferaw, Max Y. Jin, Alaa Abd-Elsayed

**Affiliations:** 1 Anesthesiology and Pain Department, Fondazione Istituto “G. Giglio”, Cefalù, Palermo, Italy; 2 University of Wisconsin School of Medicine and Public Health, Madison, WI USA; 3 Department of Anesthesiology, University of Wisconsin, Madison, WI USA; 4 University of Wisconsin-Madison, 600 Highland Ave, Madison, WI 53792 USA

**Keywords:** Epidural injections, Contrast spread patterns, Pain management, Educational review

## Abstract

**Purpose of Review:**

The purpose of this educational review is to describe the contrast spread patterns that indicate accurate needle placement in the epidural space and spread patterns associated with erroneous needle insertion.

**Recent Findings:**

Epidural injections are minimally invasive and commonly used for patients with acute and chronic back pain that does not respond to conservative management. Imaging with contrast is frequently used during this procedure to improve accuracy and reduce adverse events. Contrast spread patterns are an important tool that can help identify where the needle is placed and whether the placement is accurate. Despite this, there may be discrepancies in the interpretation of spread patterns which ultimately reduce the utility of contrast. Inaccurate needle placement may result in intrathecal/subarachnoid, subdural, fascial, or retrodural space of Okada injections.

**Summary:**

The correct interpretation of contrast spread patterns on imaging is crucial for confirming accurate epidural needle placement. Furthermore, understanding contrast patterns of improper needle placement can prevent adverse events that result from injection outside of the epidural space.

## Introduction

Low back pain is a common issue, with an incidence ranging from 6.3 to 15.4%, and a 24–80% recurrence rate within a one year period [1]. It has been shown that the prevalence of chronic back pain affects 21–68% of individuals at the age of 60 and is predicted to increase in prevalence in future years [2]. Low back pain also affects an individual’s ability to attend work, leading to indirect costs ranging from $100 to $200 billion [3].

Common causes of chronic low back pain include osteoarthritis, cervical and lumbar facet arthropathy, spondylolisthesis, spinal stenosis, discogenic pain, and other types of radicular pain [4, 5]. Conservative management of chronic lower back pain includes the use of acetaminophen, non-steroidal anti-inflammatory drugs (NSAIDs), exercise, physical therapy, opioids, antidepressants, and manipulative therapy [6]. Pain that is refractory to these measures can often benefit from more invasive therapies including steroid injections, radiofrequency ablations, and surgeries [7].

Epidural injections are a minimally invasive and commonly practiced procedure used in the treatment of acute and chronic back pain. Epidural injections work via neuraxial pain control by blocking sensory and motor spinal nerve roots in the epidural space. There are different approaches to administering into the epidural space, including the landmark-based approach, loss-of-resistance technique, caudal epidural block, ultrasound-guided epidural anesthesia, and medial and paramedian approaches. The most common techniques to access the epidural space are the transforaminal, interlaminar, and caudal approach [5].

Epidural injections are often performed without imaging and rely solely on anatomic landmarks for accurate delivery. However, imaging with contrast is commonly used to assist needle placement, ensure accurate delivery, and avoid puncturing tissues or structures outside the epidural space. The use of contrast increases the accuracy of placement and improves the outcomes of procedures by minimizing errors from improper needle placement [8]. Contrast also allows for the examination of spread patterns to ensure proper location. Despite the use of contrast, some improper placement of the needle, contrast, and pharmacotherapy may be attributable to discrepancies in the interpretation of contrast spread patterns.

This educational review identifies the contrast spread patterns associated with proper needle placement and discusses the common spread patterns of improper needle placement.

## Fluoroscopic Contrast Patterns

Needle tip placement is fundamental to successful epidural placements, and characteristic fluoroscopic patterns can be seen based on needle placement. Optimal placement of the epidural needle commonly follows characteristic findings on anterior-posterior (AP) and contralateral oblique (CLO) fluoroscopy. Before injection of contrast, the interlaminar space is identified on AP imaging, and appropriate depth is approximated on CLO and lateral imaging.

### Epidural Contrast Patterns

**Fig. 1:**
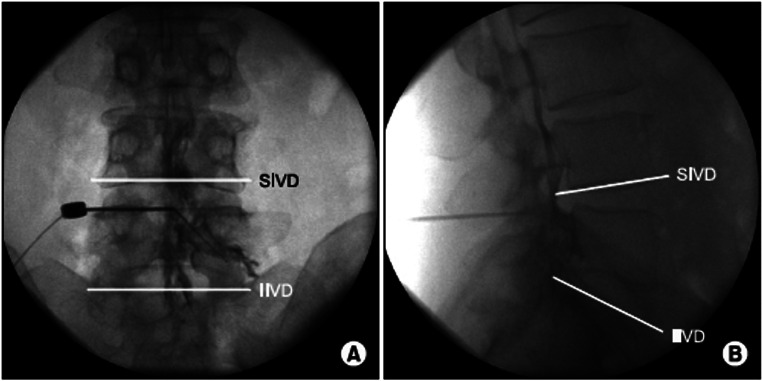
Anterior-posterior (**A**) and lateral (**B**) views of epidural steroid injection with contrast localized to the epidural space. (Image courtesy of Giuliano Lo Bianco, MD)

When contrast is correctly injected in the epidural space, asymmetric and often unilateral spread of contrast localized between the medial aspect of the pedicle and along the anteromedial aspect of the lamina can be visualized on the AP view (Fig. 1**)**. Additionally, fat vacuolization can be in the AP view, which is a reassuring but not definitive finding seen in optimal epidural placement. On CLO imaging, contrast can be seen traveling the ventral to the lamina along the ventral inter-laminar line (VILL) in optimal epidural placement [9, 10].

### Intrathecal/Subarachnoid Contrast Patterns

**Fig. 2:**
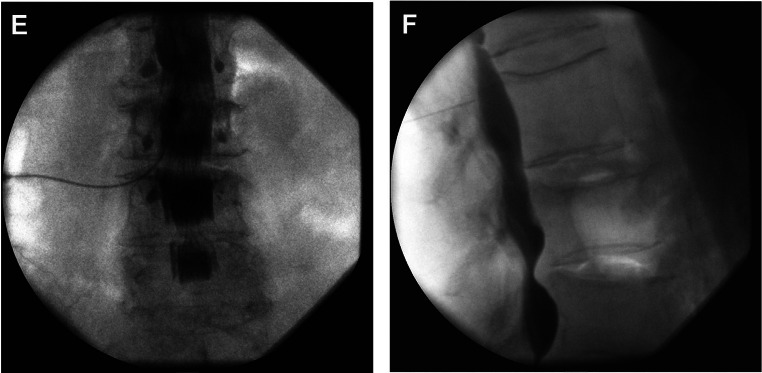
Anterior-posterior (**E**) and lateral (**F**) fluoroscopy with an intrathecal spread of contrast. (Image courtesy of Giuliano Lo Bianco, MD)

Intrathecal injection of contrast also follows characteristic patterns that can be visualized on AP and CLO imaging fluoroscopy. In the AP view, the contrast will often localize medially near the midline and be distributed symmetrically within the intrathecal space (Fig. 2). Additionally, in comparison to epidural contrast, the contrast will not be visualized along the medial border of the lamina as the contrast is likely confined to the intrathecal space [9]. On CLO, contrast will not be seen along the VILL, and there is a CSF-contrast fluid-fluid level with contrast located ventrally within the space [9, 10].

### Subdural Contrast - Localized Spread

**Fig. 3:**
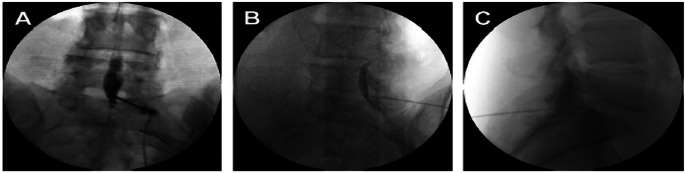
Anterior-posterior (**A**) lateral (**B**), and CLO (**C**) fluoroscopy with subdural spread of contrast with sharp margins localized to the dorsal aspect of the subdural space. (Image courtesy of Giuliano Lo Bianco, MD)

Subdural injection of contrast classically presents along two typical patterns. If the contrast is localized to the dorsal aspect of the subdural space, on an AP view, the contrast will show a rounded mass with sharp and distinct margins where thecal layers have not cleaved and a contrast pattern that does not communicate with the medial pedicle on AP or the VILL in the CLO view (Fig. 3) [11].

### Subdural Contrast - Bilateral Spread

**Fig. 4:**
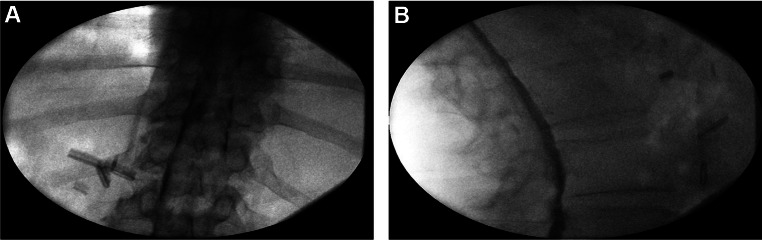
Anterior-posterior (**A**) and lateral (**B**) fluoroscopy with subdural spread of contrast with linear “tram track” pattern along the dorsal aspect of the subdural space. (Image courtesy of Giuliano Lo Bianco, MD)

Additionally, contrast injected into the subdural space may also spread with symmetric and bilateral linear patterns along the dorsal sac associated with a classic “tram track” pattern identifiable in AP view (Fig. 4) [11]. In the lateral view, the contrast can be visualized in thin linear columns traveling cephalad with contrast visible in the dorsal subdural space [9, 11].

### Fascial Spread

**Fig. 5:**
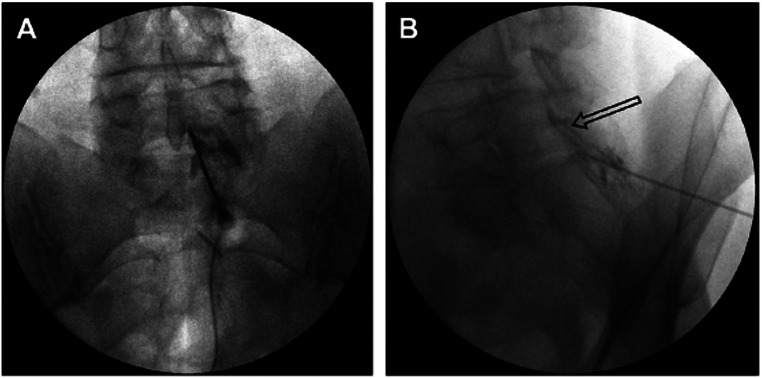
Anterior-posterior (**A**) and CLO (**B**) fluoroscopy with fascial spread of contrast localized dorsal to the VILL. (Image courtesy of Giuliano Lo Bianco, MD)

The contrast in the extradural space with fascial spread is localized with limited caudal or rostral spread and does not reach the medial pedicle, as is often readily identifiable in the AP view (Fig. 5). On the CLO view, the contrast will be dorsal to the VILL and localized to that region [9, 11].

### Retrodural Space of Okada

**Fig. 6:**
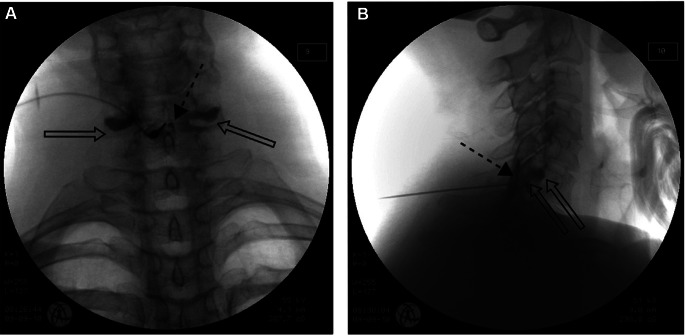
Anterior-posterior (**A**) and lateral (**B**) fluoroscopy with contrast in the retrodural space of Okada with spread visualized with characteristic bilateral communication of fluid and contrast into the facet joints through the retrodural space of Okada. (Image courtesy of Giuliano Lo Bianco, MD)

Contrast spread into the retrodural space of Okada presents a very classic pattern in the AP view. The retrodural space of Okada is a potential space that communicates between the bilateral facet joins of a corresponding level and is often present with co-existing pars interarticularis defect (Fig. 6). When contrast is injected into this space, it demonstrates contrast spread at the facet joints bilaterally with communication facilitated by the retrodural space of Okada, and this finding can be readily identified on the AP view [12].

## Epidural Complications

Due to the location and surrounding anatomic tissues, complications with epidural injections can arise. The risks associated with epidural injections are often categorized into immediate and delayed adverse effects. A multi-institutional study investigating adverse effects in epidural steroid injections. The most common immediate adverse event was vasovagal reaction 1.2% [13]. Other immediate adverse events include aborted procedures 0.8%, ED transfer < 1%, and dural puncture < 1% [13, 14]. Delayed adverse events included increased pain 2.1%, central steroid effect 2.6%, CSF leak/spinal headache < 1%, diabetic complication < 1%, and allergic reaction < 1% [13, 14]. Other adverse events reported in the literature include neural injury 0.006%, post-dural puncture headache (PDPH) up to 75%, cauda equina syndrome 0.0027%, epidural catheter infections 0.03 − 0.13%, epidural hematoma, and air emboli [14].

## Incidence and Clinical Implications of Improper Needle Placement

Expected clinical outcomes with inadvertent injection not in the epidural space are dependent on the location of the needle and may be predicted by contrast spread patterns. For correctly placed epidural injections, pain improvement can be expected as the medication is administered in the desired anatomical location. Inadvertent injection into the subarachnoid space has been reported in up to 1.2% of injections [15]. Subarachnoid injections are associated with spinal headache, paresthesias, cauda equina syndrome, conus medullaris syndrome, arachnoiditis, or meningitis due to dural puncturing and irritation within the intrathecal space [9]. Injection into the subdural space has been reported in up to 1.6% of injections, may induce a mass effect and cause neural compression resulting in pain, neural irritation, sensory or motor blockade, and autonomic nervous system effects up to and including respiratory paralysis and hemodynamic instability [9, 15]. The incidence of injections into the retrodural space of Okada has been reported in the range of 2.9% to 6.0 in the cervical spine and 0.6–7.5% of epidural injections of the lumbar spine and commonly presents as a false loss of resistance and subsequent improper injection into the retrodural space of Okada and not the epidural space proper resulting in decreased efficacy [16, 17].

## Imaging in Epidural Injections

Fluoroscopy is commonly used as the standard imaging for epidural placement with its ability to visualize the bony structures of the spine and confirm needle location and depth before injection of medication [18]. Fluoroscopy imaging is typically performed with a c-arm and with AP, lateral, and CLO imaging used to confirm needle location and depth. Fluoroscopy involves the risk of radiation exposure during imaging, which has allowed ultrasound to become an increasingly common imaging modality for epidural injections [19].

Ultrasound is an increasingly common imaging modality that may offer additional benefits and is equally effective as fluoroscopy in several large randomized controlled trials [18–21]. A prospective randomized clinical trial by Yang et al. 2016 investigated 80 patients randomized to ultrasound or fluoroscopy and showed no significant differences in pain or adverse events. However, patients with BMI > 30 were excluded from this study due to excess fat, which may obscure ultrasound imaging quality and may be suggestive of the continued utility of fluoroscopy [19]. Ultrasound has been able to identify vessels in the anterior and posterior foraminal space, which may offer the ability to prevent intravascular injections or vascular injury [18–21]. Ultrasound has been shown to have shorter operation times, decreases radiation exposure, and no significant difference in pain relief compared to fluoroscopy [19]. There is some evidence to demonstrate that ultrasound-guided epidural steroid injections decrease intravascular events; however, larger prospective randomized studies are needed to further investigate these findings [20].

## Assistive Devices in Epidural Injections

Before the injection of contrast and examination of the subsequent spread pattern, the loss of resistance (LOR) technique is used to determine when the spinal needle enters the epidural space. Although it is the most commonly used technique for epidural access, failure rates can remain high. Assistive devices that aid in the loss of resistance technique are commonly used in epidural placement to reduce failure rates during the procedure. Assistive devices that are effective in improving epidural outcomes include but are not limited to loss of resistive devices, acoustic devices, and epidural balloons, which have been found to reduce the inability to locate the epidural space, reduce the risk of complications, and are more effective than traditional air or saline LOR techniques [22]. Loss of resistance devices has been shown to have lower failure rates and decrease the time to identify the epidural space compared to traditional LOR technique [23]. These assistive devices may continue to increase in use; however, the experience and technical skill of the administering operator is still an important factor as these devices increase in use.

## Conclusion

Epidural injections are a widely used and effective treatment for acute and chronic back pain. A thorough understanding of imaging and contrast patterns is fundamental for successful epidural placements. Identification of contrast patterns associated with improper placement and their clinical implications is increasingly important as imaging and contrast guidance injections play a crucial role in many epidural injections.

## Data Availability

No datasets were generated or analysed during the current study.
